# Options for the recovery of mental activity in children after acute brain damage

**DOI:** 10.1192/j.eurpsy.2024.330

**Published:** 2024-08-27

**Authors:** Y. Sidneva, A. Zakrepina, S. Valiullina, M. Bratkova

**Affiliations:** ^1^Rehabilitation, Clinical and Research Institute of Emergency Pediatric Surgery and Trauma; ^2^Neuropsychiatric research, N.N.Burdenko National Medical Research Center of Neurosurgery; ^3^Defectology, Federal state budgetary scientific institution “Institute of correctional pedagogy”; ^4^Moscow City University, Moscow, Russian Federation

## Abstract

**Introduction:**

Children with acute brain damage make up a large group of patients who require multi-stage rehabilitation. Rehabilitation requires the creation of special conditions for psychiatric care and psychological and pedagogical correction of the consequences of severe damage to the nervous system. A differentiated approach to rehabilitation will help restore mental activity with greater efficiency, and subsequently adapt the child to the familiar environment.

**Objectives:**

**The aim** of the study is to identify the options for mental activity during the restoration of the level of consciousness in children after acute severe brain damage.

**Methods:**

210 children under the age of 18 with severe brain damage (traumatic brain injury, hypoxia, hydrocephalus), admitted for treatment and rehabilitation. Clinical-psychopathological, pedagogical methods were used; additionally - diagnostic scales, questionnaires.

**Results:**

Depending on the level of consciousness, mental activity, 4 groups were formed:

1st group - 37 (18%) patients had manifestations of mental activity with physical, cognitive and social capabilities in the minimal consciousness “+” (a- / hyperkinetic mutism with emotional reactions, understanding of addressed speech);

2nd - 67 (32%) patients had manifestations of physical and cognitive abilities with minimal consciousness “-” (a- / hyperkinetic mutism without emotional manifestations and understanding of addressed speech);

3rd - 95 (40%) patients had only the manifestation of physical capabilities at the exit from the vegetative status.

4th - 11 (10%) patients had a low manifestation of mental activity in the form of physical capabilities with a vegetative status.

**Conclusions:**

4 variants of mental activity in children after acute severe brain damage have been identified: from minimal involuntary reactions or their absence in vegetative status to voluntary actions according to the instructions of an adult in minimal consciousness “+”. Taking into account the variability of mental activity helps to differentiate the methods of psychiatric and psychological-pedagogical assistance in the recovery of children already in the early stages of rehabilitation.

**Disclosure of Interest:**

None Declared

